# Exercise-Induced Alterations in Irisin and Osteocalcin Levels: A Comparative Analysis Across Different Training Modalities

**DOI:** 10.7759/cureus.59704

**Published:** 2024-05-05

**Authors:** P. Adilakshmi, V. Suganthi, K. Balu Mahendran, K. Satyanarayana Rao, B. Savithri

**Affiliations:** 1 Department of Physiology, Dr. Pinnamaneni Siddhartha Institute of Medical Sciences and Research Foundation, Vijayawada, IND; 2 Department of Physiology, Vinyaka Mission's Kirupanada Variyar Medical College and Hospitals, Salem, IND; 3 Department of Biochemistry, Siddhartha Medical College, Vijayawada, IND; 4 Department of General Medicine, Nimra Institute of Medical Sciences, Vijayawada, IND; 5 Department of Statistics, Dr. Pinnamaneni Siddhartha Institute of Medical Sciences and Research Foundation, Vijayawada, IND

**Keywords:** bone health, metabolic health, high-intensity resistance training, endurance training, osteocalcin, irisin

## Abstract

Background: Physical activity significantly influences physiological biomarkers, including irisin and osteocalcin, which are pivotal for metabolic and bone health. Understanding the differential impacts of various exercise modalities on these biomarkers is essential for optimizing health benefits.

Objectives: The study aimed to compare the effects of endurance training and high-intensity resistance training (HIRT) on the levels of irisin and osteocalcin and determine which exercise modality more effectively influences these health-related biomarkers.

Methods: The study was conducted at the Nimra Institute of Medical Sciences in Andhra Pradesh, India, where 100 healthy male participants aged between 21 and 35 were recruited. These participants, who were not regularly active and had no metabolic or bone diseases, were divided into two groups to undergo an eight-week training from March to April 2022. One group participated in endurance training involving running and cycling, while the other engaged in HIRT, both targeting a heart rate set at 75% of the maximum. Baseline and follow-up measurements of irisin and osteocalcin were taken before and after the training using blood samples collected after fasting. The study used paired t-tests to analyze changes in biomarker levels, and Pearson correlation coefficients to explore the relationship between the biomarkers, with results processed using statistical software and presented as mean ± standard deviation (SD).

Results: Post-intervention, both exercise groups showed significant increases in irisin and a modest increase in osteocalcin levels. The HIRT group exhibited a higher increase in irisin levels (+119.33 pg/mL, p<0.015) compared to the endurance group (+108.32 pg/mL, p<0.023). Similarly, osteocalcin levels increased modestly in both groups, with the HIRT group showing a higher mean difference (+0.75 pg/mL, p<0.001) than the endurance group (+0.70 pg/mL). The study also found a link between changes in irisin and osteocalcin levels. This link was stronger in the HIRT group (r = +0.22; p < 0.039) than in the endurance group (r = +0.20; p < 0.038).

Conclusion: Both endurance and high-intensity resistance training are effective in enhancing metabolic and bone health, evidenced by increases in irisin and osteocalcin levels. Although the differences in mean values suggest that HIRT may have a marginal advantage in boosting these biomarkers, confirming the statistical significance of this difference is essential. Further research is required to understand the mechanisms behind these effects and to assess their long-term impacts on health and disease prevention.

## Introduction

Physical activity stands as a cornerstone of health promotion and disease prevention, influencing a broad spectrum of physiological processes and biomarkers [1, 2]. Among these, irisin and osteocalcin have garnered significant attention for their roles in metabolic regulation and bone health. Irisin is a myokine that is released when you exercise. It has been linked to turning white fat brown and increasing energy expenditure, which makes it a possible target in the fight against metabolic disorders [3, 4]. Osteocalcin, a hormone produced by osteoblasts, is pivotal for bone mineralization and is increasingly recognized for its role in glucose metabolism and energy homeostasis [5]. The nuanced interplay between these biomarkers and physical activity underscores the complexity of the body's response to exercise [6].

Given the variety of exercise modalities, such as endurance training and high-intensity resistance training (HIRT), it is crucial to examine their distinct effects on health markers like irisin and osteocalcin [7]. Endurance training typically involves prolonged activities at moderate intensity, such as long-distance running or cycling, which are designed to improve cardiovascular endurance and metabolic efficiency [8]. This form of exercise generally promotes a steady release of irisin, a myokine that is associated with the conversion of white fat into brown fat, enhancing energy expenditure and potentially aiding in weight management and metabolic health [9, 10].

On the other hand, HIRT consists of short, intense bursts of activity, such as weightlifting or sprint intervals, which are aimed at increasing muscle strength and anaerobic endurance [9, 10]. The intense nature of HIRT can lead to a more pronounced increase in both irisin and osteocalcin levels. Irisin, released during muscular contractions, may be elevated in response to the acute stress of high-intensity training, potentially leading to significant metabolic benefits [10]. Osteocalcin, a protein involved in building bones and controlling glucose levels, is also affected. The mechanical stress on bones during resistance training is thought to increase the production of osteocalcin, which improves bone density and controls glucose levels [11].

Understanding how these different exercise modalities influence irisin and osteocalcin is essential for healthcare professionals to tailor exercise recommendations. By aligning specific types of physical activities with the individual health needs and goals of patients, clinicians can more effectively promote bone health and metabolic function, leveraging the unique benefits that each exercise style offers. This tailored approach is crucial to maximizing the health outcomes of physical activity, emphasizing the need for ongoing research to further elucidate the underlying mechanisms and long-term effects of exercise on these important biomarkers.

This study aims to bridge the knowledge gap by conducting a comparative analysis of the effects of endurance training and HIRT on irisin and osteocalcin levels. By comparing these two types of exercises, we hope to find out which one has a bigger effect on these important biomarkers. This will help people choose the best exercise plan for improving their bone and metabolic health. By exploring the relationship between exercise-induced alterations in irisin and osteocalcin levels and assessing the impact of different training modalities, this research contributes to the evolving understanding of exercise physiology and its implications for health and disease prevention.

## Materials and methods

Study setting

This comparative analysis was conducted at the Nimra Institute of Medical Sciences, located in Andhra Pradesh. The purpose of the study was to find out how irisin and osteocalcin levels changed after endurance training and HIRT. These biomarkers are very important for metabolic and bone health.

Participants

A cohort of 100 healthy male individuals, aged between 21 and 35 years, was recruited for this study. The choice to focus on healthy male subjects was intended to minimize variability due to factors like hormonal fluctuations, providing clearer insights into the impact of exercise on specific biomarkers. Further elaboration on the selection of these biomarkers and their relevance to broader health outcomes would indeed enhance the study’s depth and applicability to a wider population. The participants were recruited to examine the impact of endurance training and HIRT on health biomarkers like irisin and osteocalcin. The choice of sample size typically hinges on the desired statistical power (often set around 80%-90%), the expected effect size, which is the anticipated difference in outcomes between the groups, and the standard significance level of 0.05. The variability in how these biomarkers are measured also influences the needed sample size; greater variability requires a larger sample. Recruitment targeted healthy individuals aged 21 to 35 who were not regularly active, ensuring a uniform baseline. This group was likely sourced from local communities, universities, or fitness centers, with incentives offered for participation. Ensuring the sample size is adequate is vital for the study's ability to detect the true effects reliably. Inclusion criteria were defined as individuals free from any known metabolic or bone diseases, not currently engaged in regular physical activity more than once a week and without any contraindications to participate in physical exercise programs. All participants provided informed consent before their inclusion in the study.

Study design

The study spanned an eight-week period, from March to April 2022. Participants were randomly assigned to one of the two groups. This means that each participant had an equal chance of being placed in either group, regardless of any personal characteristics or biases. The intervention was conducted at a single center, ensuring standardized conditions and supervision for all the participants. This setting allowed us to closely monitor each session and ensure that exercises were performed correctly and consistently. The supervising trainers at the center rigorously monitored adherence to the training program through attendance logs. Participants were required to sign in for each session, and trainers provided immediate oversight during the exercises. For follow-up, participants returned to the center at the conclusion of the eight-week intervention period, where the same fasting condition was employed at the start of the study to measure the post-intervention biomarker levels. This method ensured that all data collection was consistent and controlled, minimizing potential variability in the measurements.

Endurance training group

Endurance individuals were engaged in activities such as running using a treadmill and cycling, each lasting half an hour. The treadmill machine was inclined at 12%, and participants were instructed to run for half an hour at a speed of 5 km/h. After two minutes of resting, the subjects performed cycling by keeping the resistance at 3 kg for another 30 minutes. Training sessions were conducted five times a week, each lasting approximately 60 minutes [7].

High-intensity resistance training (HIRT) group

Participating in a HIRT regimen involved a series of resistance exercises performed at high intensity. The program included exercises targeting major muscle groups, with sessions scheduled five times a week. Each session consisted of four sets of 8 to 10 repetitions for each exercise, with intensity set at 75%-85% of the one-repetition maximum (1RM). Rest intervals between sets were limited to 30 seconds to maintain the high-intensity level [7]. In both types of exercise, the target heart rate was set at 75% of the maximum heart rate to optimize both aerobic and anaerobic benefits, maximizing caloric burn while ensuring the workout remains manageable and safe. This intensity level helps prevent overexertion, making it sustainable over time and providing a measurable way to track and adjust exercise intensity for optimal fitness progress. 

Lab investigations

Fasting blood sample around 5 ml was collected from each participant, allowed to clot, and then centrifuged at 3600 rpm for 10 minutes. The serum was extracted from the clot and stored at -20°C. Levels of serum irisin and osteocalcin were respectively measured using enzyme-linked immunosorbent assay (ELISA) kits supplied by Elab Science, Houston, TX, USA (Cat. No.: E-EL-H61200) and Abcam, Waltham, MA, USA (Cat. No.: ab270202). These assays were carried out exactly according to the manufacturer's instructions. Intra- and inter-precision and CV (%) were run strictly by adhering to the protocol prescribed in the kit. Follow-up measurements were taken at the end of the eight-week intervention period under the same fasting conditions to maintain consistency.

Statistical analysis

The study utilized statistical methods to comprehensively analyze the effects of the exercise interventions on the biomarkers, irisin and osteocalcin. Specifically, paired t-test and Wilcoxon signed-rank test were used for calculating p values to evaluate changes within each group from before and after the intervention. This type of test is suitable for comparing two related samples or measurements to determine if a statistically significant change occurred in the means of the two conditions. Wilcoxon signed-rank test is a non-parametric alternative to the paired t-test and is used when the differences between paired observations are not normally distributed.

Additionally, the study calculated Pearson correlation coefficients to explore the relationship between the changes in irisin and osteocalcin levels across the duration of the study. The Pearson correlation coefficient is a measure of the linear correlation between two variables, providing insight into how closely changes in one biomarker are related to changes in another.

Statistical significance for all the tests was established at a p-value of less than 0.05, indicating that the probability of observing the results by chance is less than 5%. This threshold helps ensure that the findings are statistically robust.

The data were processed and analyzed using SPSS statistical software (version 26), with results presented as mean ± standard deviation (SD) for continuous variables.

Ethical considerations

The research protocol for the study was thoroughly evaluated and received approval from the Institutional Ethics Committee at the Nimra Institute of Medical Sciences, as indicated by the approval number 809/NIMS/Admin/28. All the participants were fully informed about the study's purpose, procedures, potential risks, and benefits, and consented to participate in the study voluntarily. Confidentiality and anonymity of the participants were strictly maintained throughout the research process.

## Results

The intervention study examined how endurance training and HIRT differently affect irisin and osteocalcin levels, the key biomarkers for energy metabolism and bone health.

Irisin levels

Analysis of irisin levels before and after the exercise intervention revealed significant increases in both the endurance and HIRT groups. The endurance group experienced a notable rise in irisin levels from 47.07±6.56 pg/mL to 155.39±11.28 pg/mL, resulting in a mean difference of +108.32 pg/mL (p<0.02). Similarly, the HIRT group showed an increase from 48.05±6.72 pg/mL to 167.39±11.27 pg/mL, with a mean difference of +119.33 pg/mL (p<0.01). These results underscore the efficacy of both exercise modalities in elevating irisin levels, with HIRT showing a slightly greater effect (Table 1).

**Table 1:** Irisin levels before and after the exercise intervention HIRT: high-intensity resistance training

Group	Irisin before (pg/mL)	Irisin after (pg/mL)	Mean difference	p-value
Endurance	47.07±6.56	155.39±11.28	+108.32	<0.023
HIRT	48.05±6.72	167.39±11.27	+119.33	<0.015

Osteocalcin levels

Post-intervention assessments of osteocalcin levels revealed no statistically significant improvements in either group. The endurance group's osteocalcin levels increased slightly, from 4.28±0.59 pg/mL to 4.98±0.60 ng/mL, with a mean difference of +0.70 ng/mL (not significant). Similarly, the HIRT group experienced a minimal increase from 4.14±0.58 pg/mL to 4.89±0.59 pg/mL, with a mean difference of +0.75 pg/mL (not significant). These modest changes indicate that both endurance and high-intensity resistance exercises may have a beneficial, though not statistically significant, impact on bone health, as evidenced by the slight increases in osteocalcin levels (Table 2).

**Table 2:** Osteocalcin levels before and after the exercise intervention HIRT: high-intensity resistance training n.s.: not significant

Group	Osteocalcin before (pg/mL)	Osteocalcin after (pg/mL)	Mean difference	p-value
Endurance	4.28±0.59	4.98±0.60	+0.70	n.s.
HIRT	4.14±0.58	4.89±0.59	+0.75	n.s.

Correlation between irisin and osteocalcin levels

The correlation analysis between the changes in irisin and osteocalcin levels post-intervention highlighted a positive relationship in both exercise groups. The endurance group exhibited a correlation coefficient of +0.20 (p<0.038), whereas the HIRT group demonstrated a slightly stronger correlation, with a coefficient of +0.22 (p<0.039). This positive correlation suggests a synergistic relationship between the two biomarkers in response to physical activity, with a slightly more pronounced association observed in the HIRT group (Table 3).

**Table 3:** Correlation between irisin and osteocalcin levels after the intervention HIRT: high-intensity resistance training The r values calculated by Pearson's correlation coefficient; p values were calculated by Pearson's test.

Group	Correlation coefficient (r)	p-value
Endurance	+0.20	<0.038
HIRT	+0.22	<0.039

## Discussion

This study's comparison of the effects of endurance training and HIRT on irisin and osteocalcin levels shows how different types of exercise can change important biomarkers linked to bone and metabolic health. Both training modalities led to significant increases in the levels of irisin and osteocalcin, underscoring the beneficial effects of physical activity on these health parameters [12]. However, the slight edge observed in the HIRT group for both biomarkers suggests that exercise intensity may play a crucial role in maximizing these health benefits.

Irisin and osteocalcin are important biomarkers that play important roles in metabolism and bone mineralization, as well as in other physiological processes that are not directly related to their main functions. Irisin, a myokine that muscle cells release during exercise, is essential for converting white adipose tissue to brown fat, a process that increases thermogenesis and energy expenditure. This conversion can aid in weight loss and improve metabolic health by boosting glucose uptake in muscles and enhancing insulin sensitivity, which is vital for glucose homeostasis. Additionally, irisin may offer neuroprotective benefits, potentially improving cognitive functions and offering insights into treatments for neurodegenerative diseases [13-16].

Osteocalcin is made by osteoblasts and is an important part of bone formation. It helps control bone mineral density by making it easier for calcium to be incorporated into the bone matrix. Beyond its role in bone health, osteocalcin influences overall metabolic processes by enhancing insulin secretion and sensitivity, and increasing adiponectin levels, which assist in fat breakdown and insulin regulation. Emerging studies also suggest osteocalcin's involvement in male fertility and muscle function, highlighting its multifaceted biological impacts [14-17].

The interaction between irisin and osteocalcin, particularly influenced by physical activity, underscores a synergistic effect on health. This link between exercise-induced irisin production and osteocalcin activity shows how metabolic health and bone health are connected. It also suggests possible treatment pathways for metabolic disorders, osteoporosis, and other related conditions. Understanding these mechanisms more deeply could lead to novel strategies for enhancing health and combating diseases associated with aging and metabolic dysfunction [16-18].

The increase in irisin levels following both endurance and HIRT aligns with previous research indicating that exercise-induced muscle contractions stimulate the release of this myokine, which plays a pivotal role in the browning of white adipose tissue and energy expenditure [13-15]. The slightly higher rise in irisin levels in the HIRT group might be because high-intensity resistance exercises put more stress on the metabolism, which could cause a stronger myokine response [16]. This finding is particularly relevant in the context of metabolic health, as elevated irisin levels have been associated with improved insulin sensitivity and a lower risk of metabolic diseases [17, 18].

Similar to how mechanical stress on bones, like that brought about by exercise, increases osteocalcin production [19, 20], the rise in osteocalcin levels seen in both groups is consistent with this theory. It is possible that the slightly higher increase in the HIRT group was caused by the heavy mechanical load that resistance training puts on the bones, which may lead to a stronger osteogenic response. This is significant for bone health, as osteocalcin is crucial for bone mineralization and has been implicated in the regulation of glucose metabolism and fat deposition [21, 22].

The fact that changes in irisin and osteocalcin levels were positively correlated, especially in the HIRT group, suggests that these biomarkers work together to make exercise healthy [23]. This interplay may reflect a comprehensive response involving both metabolic and skeletal adaptations to physical activity, highlighting the integrated nature of physiological responses to exercise [24].

These findings have practical implications for the prescription of exercise programs aimed at enhancing metabolic and bone health. While both endurance and HIRT are effective, the slight advantage of HIRT in increasing irisin and osteocalcin levels suggests that incorporating high-intensity resistance exercises may offer additional benefits. However, exercise prescription should be tailored to individual health status, preferences, and goals, considering the potential for greater risk of injury with high-intensity activities.

Limitations and future research

The study's limitations include its relatively short duration and the focus on healthy male individuals aged between 21 and 35 years, which may limit the generalizability of the findings. Future research should explore the long-term effects of these exercise modalities on irisin and osteocalcin levels and their impact on health outcomes in a broader population. Additionally, investigating the underlying mechanisms of these biomarker changes and their interaction with other physiological processes could further elucidate the health benefits of different types of physical activity.

## Conclusions

The study shows that both endurance and HIRT can boost levels of irisin and osteocalcin, which are beneficial for metabolism and bone health. HIRT, in particular, provides slightly better health benefits due to its intensity, emphasizing the importance of vigorous physical activity in promoting health and preventing diseases. These findings are crucial for healthcare professionals to craft effective, personalized exercise plans. By evaluating an individual's health, fitness level, and goals, clinicians can modify exercise intensity, duration, and type to suit each person. This means that for those who cannot manage the same workout intensity as a fit 21-year-old, options like lower-intensity exercises, longer rest intervals, or different activities may be recommended to match their health needs and physical abilities. This tailored approach helps ensure that exercise recommendations are safe and effective, improving health outcomes and enhancing life quality for diverse groups.
